# Classification of high dimensional biomedical data based on feature selection using redundant removal

**DOI:** 10.1371/journal.pone.0214406

**Published:** 2019-04-09

**Authors:** Bingtao Zhang, Peng Cao

**Affiliations:** 1 School of Electronic and Information Engineering, Lanzhou Jiaotong University, Lanzhou, China; 2 Key Laboratory of Opto-technology and Intelligent Conrtol Ministry of Education, Lanzhou Jiaotong University, Lanzhou, China; 3 School of Architecture and Urban Planning, Lanzhou Jiaotong University, Lanzhou, China; Northeast Normal University, CHINA

## Abstract

High dimensional biomedical data contain tens of thousands of features, accurate and effective identification of the core features in these data can be used to assist diagnose related diseases. However, there are often a large number of irrelevant or redundant features in biomedical data, which seriously affect subsequent classification accuracy and machine learning efficiency. To solve this problem, a novel filter feature selection algorithm based on redundant removal (FSBRR) is proposed to classify high dimensional biomedical data in this paper. First of all, two redundant criteria are determined by vertical relevance (the relationship between feature and class attribute) and horizontal relevance (the relationship between feature and feature). Secondly, to quantify redundant criteria, an approximate redundancy feature framework based on mutual information (MI) is defined to remove redundant and irrelevant features. To evaluate the effectiveness of our proposed algorithm, controlled trials based on typical feature selection algorithm are conducted using three different classifiers, and the experimental results indicate that the FSBRR algorithm can effectively reduce the feature dimension and improve the classification accuracy. In addition, an experiment of small sample dataset is designed and conducted in the section of discussion and analysis to clarify the specific implementation process of FSBRR algorithm more clearly.

## Introduction

The analysis of high dimensional disease data [1–2] is a very important research field, especially cancer [3], or mental disease (e.g. Depressive [4–5]). It is unrealistic to cure these diseases completely, so early diagnosis or prevention plays an important role in the treatment related disease. However, high dimension biomedical data usually contain a large number of weak relevant or irrelevant features. If all the features are treated equally, the time complexity, spatial complexity and accuracy of the prediction can be seriously affected. Therefore, feature selection is considered to be an essential step in the diagnosis of related disease using high dimension biomedical data.

As is stated in [6], feature selection is also referred to as feature subset selection. The main purpose of feature selection is to remove irrelevant and redundant features in the classification process, while retaining the most valuable information of the original data. In other words, the objective of feature selection is to select an optimal feature subset [7] from the original feature set, which lays a good foundation for subsequent classification or learning work. As one of the important part of knowledge discovery technology, feature selection [8] can effectively improve the computing speed of subsequent prediction algorithm, enhance the compactness of the prediction model, increase the generalization ability of the corresponding model, and avoid over fitting.

Based on the above factors, feature selection has always been a hot research topic, and new achievements are constantly emerging. For example, in [9], a feature selection method based on multi-objective binary based biogeography optimization (MOBBBO) is proposed for gene selection, which combines the non-dominated sorting method and the crowding distances method into the binary based biogeography optimization (BBBO) framework. In [10], a novel feature selection method via chaotic optimization is developed to solve the problem of balance between exploration of the search space and exploitation of the best solutions. In [11], Liu et al. used a discrete biogeography based optimization (DBBO) method by integrating discrete migration model and discrete mutation model for feature selection in molecular signatures. In [12], Li et al. proposed that a feature selection algorithm based on the multi-objective ranking binary artificial bee colony to select the optimal subset from the original high dimensional data while retaining a subset that satisfies the defined objective. In addition, recent advances on feature selection can be found in [13–14], in which [14] focuses on the review of the latest research work on evolutionary computation (EC) of feature selection, and identifies the contributions of these different algorithms.

To better introduce the research status of high-throughput data or high-dimensional data based on feature selection algorithm [1, 15], the following is an overview of some representative studies in recent years. Tan et al. [1] proposed a new minimax sparse LR model for very high-dimensional feature selections, which can be efficiently solved by a cutting plane algorithm. In order to solve the problem of effectively identifying chromosome-wide spatial clusters from high-throughput chromatin conformation capture data, a population based optimization algorithm coordinates and guides the non-negative matrix factorization toward global optima was proposed in [16]. In [17], a novel feature selection method based on high dimensional model representation (HDMR) was proposed to solve the hyper-spectral image classification problem. The core idea of this method is to rank the global sensitivity index calculated via the HDMR to find the most relevant features. In order to explore and identify small clusters of spatially proximal genomic regions, Li et al. [18] proposed evolutionary computation methods to evolve and confirm functionally related genomic regions. Chen et al. [19] proposed a feature selection algorithm, which was named genetic programming (GP) with permutation importance (GPPI), to select features of high-dimensional symbolic regression (SR) using GP. Based on two typical applications of microarray analysis and target detection, Augusto et al. [20] discussed the feature selection of high-dimensional spatial data. In order to solve the problem of high-dimensional data classification, Zhang [21] proposed an improved artificial bee colony (ABC) algorithm to select the optimal feature subset. Meanwhile, to improve the convergence of ABC, the modified ABC algorithm (named OGR-ABC algorithm) introduces three modified strategies including opposite initialization, global optimum based search equations and ranking based selection mechanism.

Through the analysis of the above research, it is not difficult to find that feature selection process mainly includes two steps [22]: search strategy and evaluation criterion. Based on whether or not the classifier itself is used as feature evaluation index, evaluation criterion can be categorized into the wrapper method and the filter method. The wrapper method [23, 24] to evaluate superiority and inferiority of the optimal feature subset under the premise of the classification algorithm unchanged. Meanwhile, the corresponding classification accuracy is adopted as an index to select optimal feature subset. So the feature subset selected by the wrapper method is not universal. It is necessary to execute the feature selection process again when the classification algorithm is changed. Therefore, its time complexity is too high, especially for high dimension data, and the execution time of the algorithm may be longer. Another evaluation criterion based on the filter method [25–27], the search of feature space depends on the intrinsic correlation of the data itself rather than the classification algorithm. The filter method is increasingly attractive because of its simplicity and fast speed. Therefore this method is more popular than the wrapper method.

Four intrinsic correlation metrics are often adopted by the filter method to evaluate feature subset, including MI [28], fractal dimension [29], dependency degree [30] and distance [31]. Among them, MI is considered as the most acceptable criteria due to two major advantages [32]: (1) Measuring different relationship between nonlinear (random) variables. (2) Preserving stability of transformations in the high dimensional feature space that is invertible.

According to the above analysis, a filter feature selection method is proposed for high dimensional biomedical data based on redundant removal in this paper. Firstly, we analyze the four boundary extremes of correlation between feature and target class and the correlation between feature and feature. Based on this, two redundant criterions are proposed. And then in order to quantify the redundancy criterion, the core module based on MI is proposed: the definition of approximate redundancy feature. Finally, the algorithm implementation is given.

## Mathematical symbols and basic concepts

### Mathematical symbols

There are many mathematical symbols used in this study. To improve the readability, we list these mathematical symbols and their abbreviations in below.

*P*:a probability measure.
*F*: feature set, *F* = {*F*_1_,*F*_2_,…,*F*_*i*_,…,*F*_*n*_}.
*F*_*i*_: *F*_*i*_ = {*f*_i,1_, *f*_*i*,*2*_,…,*f*_*i*,*n*_}.
*A*_*i*_: *A*_*i*_ = *F* −{*F*_*i*_}.
*C*: class attribute, *C* = {*C*_1_,*C*_2_,…,*C*_*i*_,…,*C*_*m*_}.
*R*: the relevance between any two variables.
*R*_*i*,*j*_: the relevance between any pair of feature *F*_*i*_ and *F*_*j*_,*i*≠*j*.
*R*_*i*,*c*_: the relevance between any feature *F*_*i*_ and class attribute *C*.
*R*_max_: the maximum value of *R*_*i*,*c*_.
$\bar{R}$: the mean value of *R*_i,c_, that is $\bar{R}=\frac{1}{n}\sum_{i=1}^{n}R_{i,c}$.

### Basic concepts

For laying a base for further investigation, three basic concepts [33] (strongly relevant feature, weakly relevance feature, and irrelevant feature) used in this study are listed as follows.

Strong relevance: *F*_*i*_ is strongly relevant feature *iff* there exists P(*F*_*i*_,*A*_*i*_)>0 such that

$$P(C|F_{i},A_{i})\neq P({C|A}_{i})$$ (1)

Weak relevance: *F*_*i*_ is weakly relevant feature *iff* it is not strongly relevant (i.e. P(*C*|*F*_*i*_,*A*_*i*_) ≠ P(*C*|*A*_*i*_)), there exists $A_{i}^{\prime }\subset A_{i}$ and $P(F_{i},A_{i}^{\prime })>0$ such that

$$P(C|F_{i},A_{i}^{\prime })\neq P(C|A_{i}^{\prime })$$ (2)

Irrelevance: *F*_*i*_ is irrelevant feature *iff* it are not strongly relevant and weakly relevant, there all $A_{i}^{\prime }\subset A_{i}$ and $P(F_{i},A_{i}^{\prime })>0$ such that

$$P(C|F_{i},A_{i}^{\prime })=P(C|A_{i}^{\prime })$$ (3)

Strong relevance indicates that the feature is very important for classification accuracy improvement, so it can’t be arbitrarily removed. Weak relevance indicates that the feature can sometimes contribute to improve prediction accuracy. Irrelevance indicates that the feature is useless on the improvement of classification accuracy, so it can be directly deleted.

## Method

### Determination of redundancy criterion

It is difficult to determine that the complete correlation between any pair of feature in the actual calculation process, and then determine whether there is redundancy among features. To combat this, a redundancy criterion based on the correlation is proposed in this study to lay the foundation for further feature selection. Based on three basic concepts, the redundancy of feature *F*_*i*_ is analyzed under different extreme values of *R*_*i*,*c*_ and *R*_*i*,*j*_. Different cases of extreme value are shown in Table 1.

**Table 1:** Different cases of extreme value.

	***R***_**i,c**_	***R***_**i,j**_
**Case 1**	large	large
**Case 2**	large	small
**Case 3**	small	large
**Case 4**	small	small

It is easy to draw the following four conclusions after analyzing four cases:

Conclusion 1: *R*_*i*,*c*_ is large, which means that *F*_*i*_ contains more information about *C*. *R*_*i*,*j*_ is large, which means that the correlation between *F*_*i*_ and *F*_*j*_ is strong. If *R*_*i*,*j*_ = 1, then *F*_*i*_ and *F*_*j*_ is complete correlation, hence *F*_*i*_ is redundant. If *R*_*i*,*j*_≠1, it is difficult to determine the feature *F*_*i*_ whether or not is redundant.

Conclusion 2: *R*_*i*,*j*_ is small, which means that the correlation between *F*_*i*_ and *F*_*j*_ is weak. Hence *F*_*j*_ can’t replace *F*_*i*_. In other words, no matter the size of the *R*_*i*,*c*_, the feature *F*_*i*_ is not redundant.

Conclusion 3: *R*_*i*,*c*_ is small, which means that *F*_*i*_ contains less information about *C*. *R*_*i*,*j*_ is large, which means that the correlation between *F*_*i*_ and *F*_*j*_ is strong. In this case, the feature *F*_*i*_ is redundant with higher probability. With the increase of *R*_*i*,*j*_, this probability is also increasing.

Conclusion 4: *R*_*i*,*j*_ is small, which means that the correlation between *F*_*i*_ and *F*_*j*_ is weak. This conclusion is consistent with the conclusions 2, no matter the size of the *R*_*i*,*c*_, the feature *F*_*i*_ is not redundant.

Based on the above four conclusions, two redundant criteria can be obtained:

**Criteria 1**: when *R*_*i*,*j*_ is large, whether *F*_*i*_ is redundant is uncertain.

**Criteria 2**: when *R*_*i*,*j*_ is small, no matter the size of the *R*_*i*,*c*_, the feature *F*_*i*_ is not redundant.

### Definition approximate redundancy feature

Based on the two redundant criterions inthe previous section, the approximate redundancy feature is proposed and defined in this section.

Assuming that the *R*_*i*,*c*_ of the feature *F*_*i*_ is very close to *R*_max_, it indicates that *F*_*i*_ contains a lot of information about class attribute *C*. In this condition, only if the value of *R*_*i*,*j*_ is large enough, *F*_*i*_ can be considered as an approximate redundancy feature. Otherwise, it can’t be considered as redundancy feature. The reason is that *F*_*i*_ plays an important role in improving the accuracy of classification, and can’t be easily removed as redundancy. By contrast, Assuming that the *R*_*i*,*c*_ of feature *F*_*i*_ is not very close to *R*_max_, it indicates that *F*_*i*_ contains relatively less information about class attribute *C*. In this condition, as long as the value of *R*_*i*,*j*_ is relatively large, *F*_*i*_ is considered as an approximate redundancy feature. The reason is that *F*_*i*_ not plays a main role in improving the accuracy of classification. Based on the above analysis and discussion, the approximate redundant feature is formally described in definition 1.

**Definition 1** (approximate redundancy feature): There is any pair of correlation feature *F*_*i*_ and *F*_*j*_, and *R*_*j*,*c*≥_*R*_*i*,*c*_.

(1) *F*_*i*_ is an approximate redundancy feature *iff* there exists, *R*_max_-*R*_*j*,*c*_≤δ, 0.05≤δ≤0.13, such that

$$R_{i,j}\geq R_{max}$$ (4)

(2) *F*_*i*_ is an approximate redundancy feature *iff* there exists *R*_*max*_ − *R*_*j*,*c*_ > *δ* && *R*_*max*_ − *R*_*j*,*c*_ ≤ *α*, 0.05≤δ≤0.13, 0.60≤*α*≤0.66, such that

$$R_{i,j}>(\bar{R}+R_{j,c})/2$$ (5)

Definition 1indicates that *F*_*j*_ can be approximated as an alternative for *F*_*i*_.

### Correlation calculation

In general, correlation measure methods include linear and nonlinear. A nonlinear method based on MI is adopted in this study, and the reason is that the high dimensional biomedical data usually exist in the form of nonlinear in the real world. The correlation between any pair of variables (*X*, *Y*) can be calculated in the following formulas (6) or (7).

IG(X;Y)=H(X)−H(X|Y)=H(Y)−H(Y|X) (6)

IG(X;Y)=H(X)+H(Y)−H(X,Y) (7)

where *H*(*X*), *H*(*X|Y*) and *H*(*X*,*Y*) can be calculated on the basis of formulas (8), (9) and (10).

$$H(X)=-\sum_{i}P(x_{i})\log_{2}P(x_{i})$$ (8)

$$H(X|Y)=-\sum_{j}P(y_{j})\sum_{i}P(x_{i}{|y}_{j})\log_{2}P(x_{i}|y_{i})$$ (9)

$$H(X,Y)=-\sum_{j}P(y_{j})\sum_{i}P(x_{i},y_{j})\log_{2}P(x_{i},y_{i})$$ (10)

According on the above three formulas, the value of *H*(*X|Y*) or *H*(*X*,*Y*) is smaller when *Y* contains more information about *X*. In other words, the greater value of *IG*(*X;Y*), which means there are more relevant between *X* and *Y*.

To prevent the scale of data is not unified and to reduce the effect of extreme value, each *IG*(*X;Y*) is normalized to the range [0, 1] using formula (11).

$$R=\frac{2*IG(X;Y)}{H(X)+H(Y)}$$ (11)

### Algorithm implementation

Based on the definition of the approximate redundancy feature and the correlation calculation method, a feature selection algorithm for high dimension biomedical data classification based on redundant removal (FSBRR) is given in **Algorithm 1**.

Algorithm 1: **FSBRR**

Input: Feature set: *F* = {*F*_1_,*F*_2_,…,*F*_*i*_,…,*F*_*n*_};

        Class label: C = {C_1_,C_2_, …, C_i_…,C_*m*_};

        Parameter: τ, δ, α.

1.for *i* = 1:*n*

2. ***R***_***i*,*c***_ = **2** * ***IG***(***C***;***F***_***i***_)/(***H***(***C***) + ***H***(***F***_***i***_));

3.if ***R***_***i*,*c***_ ≥ ***τ***                % a preset threshold value, remove irrelevance features

4.Addto(*F’*, *F*_*i*_);

5.Addto(*R’*, *R*_*i*,*c*_);

6.end

7. end

8. $\bar{R}=\frac{1}{n}\sum_{i=1}^{n}R_{i,c}$;                  % the mean value of *R*_*i*,*c*_, 1≤*i*≤*n*

9. [X, I] = sort(*R*_*i*,*c*_, ‘descend’);                      % where *R*_*i*,*c*_ϵ*R’*

10.*F’* = *F’*(I);                                                % order *F’* in descending *R*_*i*,*c*_ value

11 for i = 1:size(I,2)-1;

12.*F*_*i*_ = *F’*(:,*i*); *R*_*i*,*c*_;                        % to select current first feature in each cycle, i.e. first variable

13. for j = i+1: size(I,2)

14.*F*_*j*_ = *F’*(:,*j*);                        % to select next feature (or variable)

15. ***R***_***j*,*c***_ = **2** * ***IG***(***C***;***F***_***j***_)/(***H***(***C***) + ***H***(***F***_***j***_))

16.        ***R***_***i*,*j***_ = **2** * ***IG***(***F***_***i***_;***F***_***j***_)/(***H***(***F***_***i***_) + ***H***(***F***_***j***_))

17.            if *F*_*j*_ ≠ Null

18.                if *R*_*i*,*c*_- *R*_*j*,*c*_≤δ&&*R*_*i*,*j*_≥*R*_*i*,*c*_% 0.05≤δ≤0.13

19.                    remove(*F’*, *F*_*j*_); % removing approximate redundancy features

20.else if *R*_*i*,*c*_- *R*_*j*,*c*_>δ&&*R*_*i*,*c*_- *R*_*j*,*c*_<α&&*R*_*i*,*j*_> ($\bar{R}$+*R*_*j*,*c*_)/2% 0.05≤δ≤0.13, 0.60≤α≤0.66

21.                    remove(*F’*, *F*_*j*_);

22.                end

23.            end

24.        end

25.end

26.*F*_optimal_ = *F’*;

Output: *F*_optimal_

The time consumption of FSBRR algorithm is mainly used to calculate *R*_*i*,*c*_ and *R*_*i*,*j*_, so its atomic operation is the calculation of *R*_*i*,*c*_ and *R*_*i*,*j*_. Assuming that a dataset contains *n* features, the time complexity of this algorithm used for removing irrelevant feature is linear time order O(*n*) (line 1 to line 7). For the removing approximate redundant feature and approximate irrelevant feature (line 11 to line 25): In the worst case, the time complexity is square time order O(*n*^*2*^), and all features are not redundant features at this time. In the best case, the time complexity is linear time order O(*n*), and except for the first feature, the remaining *n*-1 features are redundant features at this time.

### Performance evaluation function

In this study, classification accuracy and the number of selected features are used to design the performance evaluation function [34–35], which is shown in formula (12).

$$performance=w_{1}*Acc+w_{2}*(1-\frac{n}{N})$$ (12)

*n* is the number of selected features and *N* is the total number of features. *w*_1_ and *w*_*2*_ are predefined weight coefficients, which are used to adjust the importance of two indicators in the performance evaluation function. In this study, the values of *w*_1_ and *w*_*2*_ are set to 0.999 and 0.001 respectively. The main reason for this setting can be attributed to the following three aspects: (1) Under the prerequisite of data dimensionality reduction, this study main focuses on the use of classification accuracy as a metric of feature selection algorithm. (2) The number of selected features is significantly reduced, but the classification accuracy is not improved, such dimensionality reduction of high-dimensional data will lose its original application value. (3) The performance evaluation function with high weight coefficient of classification accuracy and low weight coefficient of the number of selected features has been recognized and widely applied in many feature selection studies, such as [34]. In addition, *Acc* is classification accuracy as defined in formula(13).

$$Acc=\frac{C_{num}}{C_{num}+I_{num}}*100\%$$ (13)

*C*_*num*_ and *I*_*num*_ are the number of correct and incorrect classification labels respectively.

## Experiments

### Data description

Eight well-known biomedical datasets (Table 2) were used to evaluate the performance of FSBRR algorithm. These dataset includes eight aspects of disease diagnosis data. The data dimension range was from 319 to 21548. The first three datasets were taken from the Kent Ridge Biomedical [36]. p53 Mutants and Arcene were taken from the UCI dataset [37]. Breast invasive carcinoma (BRCA), Glioblastoma multiforme (GBM), and tumour sequencing project (TSP) were taken from the TCGA [38].

**Table 2:** High dimension biomedical datasets.

**Dataset**	**Attributes**	**Instances**	**Classes**
**ColonTumor**	2000	62	2
**Nervous-System**	7129	60	2
**DLBCL-Stanford**	4026	47	2
**p53 Mutants**	5409	16772	2
**Arcene**	10000	200	2
**BRCA**	21548	1097	2
**GBM**	18348	528	2
**TSP**	319	163	2

### Experimental design

To evaluate the performance of FSBRR algorithm, under the same conditions, we designed and conducted the following experiments: eight high dimensional biomedical data were compared and analyzed by FSBRR, Relief (a filter methodbased on the nearest neighbor distance) [39], maximum relevance and minimum redundancy (mRmR) [40] and genetic algorithm (GA) [41], respectively. In this experiment, the same conditions contain two meanings: (1) Random forest (RF, numTrees = 10), K-nearest neighbor (KNN, k = 1), and Support Vector Machine (SVM, Linear Kernel) were adopted as classifier to evaluate classification performance. (2) In FSBRR algorithm, the parameter τ is set to 0, and the purpose is that to avoid losing the weak correlation feature without prior knowledge. In addition, after adaptive testing, the values of δ and α were set to 0.08 and 0.64 respectively.

To obtain an unbiased experimental result, 10 fold cross validation was adopted to evaluate the classification performance. Each dataset was stratified into 10 folds, of which 9 folds were used as a training sample and the remaining 1 fold was used as a testing sample. Moreover, in order to get a statistically meaningful result, each experiment was executed 100 times independently. This means that the classification task is executed 1000 times in total, and the average value is taken as the result in finally. The above experiments were implemented in Matlab 2017a. The experimental hardware and software configuration is shown in Table 3.

**Table 3:** Hardware and software configuration of experimental.

**No.**	**Components**	**Parameters**
**1**	CPU	Intel i7-8550U 4.0GHz
**2**	RAM	16G
**3**	Operating system (OS)	Windows 7
**4**	Software platform	Matlab 2017a

## Results

For the eight datasets, we have conducted the experiments described in above section. The six major statistical indicators were compared and analyzed, and the results were shown in Table 4.

**Table 4:** Experimental results based on eight data sets.

Classifier	Dataset	Algorithm	Mean (%)	Max(%)	Min(%)	Std	MeanFN	RT(s)
**RF**	ColonTumor	Full Set	78.21	82.32	71.46	3.51	2000	‒
		FSBRR	**92.01**	95.45	91.21	**1.97**	**34**	**1.04**
		Relief	85.49	89.94	81.23	3.16	38	2.19
		mRmR	86.61	90.72	82.77	3.85	42	2.75
		GA	89.13	95.71	84.12	3.91	36	19.18
	Nervous-System	Full Set	61.33	74.61	54.86	5.68	7129	‒
		FSBRR	**80.17**	84.02	74.64	**3.02**	**37**	**5.23**
		Relief	71.56	81.24	68.88	3.97	44	14.72
		mRmR	72.42	76.43	69.61	3.24	42	16.37
		GA	75.76	79.21	69.56	3.87	48	90.01
	DLBCL-Stanford	Full Set	76.60	80.21	73.79	2.87	4026	‒
		FSBRR	**82.99**	84.16	79.30	**2.33**	**29**	**2.41**
		Relief	75.48	81.64	71.63	3.34	45	3.84
		mRmR	76.58	84.61	69.15	6.42	54	4.10
		GA	79.46	82.46	75.16	3.12	91	31.80
	p53 Mutants	Full Set	89.66	92.18	81.61	3.42	5409	‒
		FSBRR	**94.31**	98.00	90.56	3.19	39	**2.49**
		Relief	90.42	94.25	84.25	4.06	54	4.14
		mRmR	87.91	91.14	79.79	5.48	**37**	4.52
		GA	94.16	98.31	91.21	**3.01**	64	37.44
	Arcene	Full Set	73.14	80.60	66.60	4.87	10000	‒
		FSBRR	**85.67**	87.30	82.20	2.11	**51**	**12.34**
		Relief	81.01	84.01	77.78	**2.06**	73	24.94
		mRmR	78.80	84.02	72.02	4.82	71	20.34
		GA	77.14	80.02	74.31	3.16	91	128.67
	BRCA	Full Set	80.57	85.51	76.99	3.29	21548	‒
		FSBRR	**86.26**	89.24	81.14	**2.51**	**148**	**18.84**
		Relief	85.22	90.10	79.44	4.01	241	26.25
		mRmR	83.54	85.92	80.36	2.21	189	24.01
		GA	85.16	88.90	81.12	2.62	246	29.60
	GBM	Full Set	69.92	80.16	62.77	8.45	18348	‒
		FSBRR	80.95	86.78	75.49	**3.95**	61	**4.56**
		Relief	76.14	82.15	70.65	4.29	**53**	4.87
		mRmR	74.90	79.12	69.44	3.96	68	5.42
		GA	**82.03**	90.25	75.02	5.48	93	12.84
	TSP	Full Set	68.77	75.87	63.21	4.26	319	‒
		FSBRR	**78.63**	81.23	76.99	**1.83**	**108**	**0.47**
		Relief	67.01	69.89	61.32	3.01	135	0.75
		mRmR	56.96	62.42	50.42	4.12	124	0.50
		GA	67.26	70.51	60.23	3.96	137	0.80
**KNN**	ColonTumor	Full Set	75.80	81.32	71.02	3.69	2000	‒
		FSBRR	**91.91**	95.01	88.09	**2.38**	**34**	**0.75**
		Relief	83.41	88.91	78.13	3.76	38	1.87
		mRmR	84.69	89.12	79.58	3.88	42	2.04
		GA	86.67	88.33	82.33	2.53	38	9.16
	Nervous-System	Full Set	56.86	69.66	53.94	4.17	7129	‒
		FSBRR	**74.24**	81.25	70.11	3.22	**37**	**2.88**
		Relief	65.69	70.89	63.28	3.36	44	9.14
		mRmR	65.77	69.12	62.14	3.28	42	8.31
		GA	73. 62	76.58	69.14	**2.93**	56	73.82
	DLBCL-Stanford	Full Set	78.24	82.46	62.36	5.83	4026	‒
		FSBRR	**83.68**	88.78	79.47	**3.42**	**29**	**1.88**
		Relief	76.32	84.47	71.95	4.73	45	2.43
		mRmR	76.98	87.71	71.54	5.86	54	3.01
		GA	80.06	82.78	72.83	3.79	97	24.31
	p53 Mutants	Full Set	84.93	90.15	80.61	4.01	5409	‒
		FSBRR	88.30	92.01	85.50	**2.09**	**39**	**2.04**
		Relief	85.20	89.23	81.99	2.45	54	3.78
		mRmR	84.27	87.50	80.09	2.29	37	4.14
		GA	**89.90**	95.14	87.04	2.99	56	39.77
	Arcene	Full Set	67.60	77.38	62.57	4.38	10000	‒
		FSBRR	**85.67**	89.30	79.20	3.02	**51**	**4.98**
		Relief	82.01	87.01	79.04	**2.89**	73	9.04
		mRmR	78.93	86.33	75.18	4.34	71	9.79
		GA	79.13	84.78	75.90	3.41	87	84.46
	BRCA	Full Set	78.51	83.42	72.81	4.87	21548	‒
		FSBRR	**84.42**	87.24	80.02	**2.71**	**148**	**10.19**
		Relief	83.75	87.06	74.59	5.23	241	19.10
		mRmR	82.43	87.88	75.31	4.85	189	20.21
		GA	83.01	87.11	78.01	3.02	251	62.62
	GBM	Full Set	68.82	80.64	65.55	4.58	18348	‒
		FSBRR	80.12	87.07	78.30	**2.87**	61	**1.85**
		Relief	74.85	80.02	70.88	3.81	**53**	2.42
		mRmR	74.82	80.25	66.40	5.56	68	2.87
		GA	**81.39**	88.51	75.24	3.95	88	5.50
	TSP	Full Set	62.72	74.87	61.21	4.86	319	‒
		FSBRR	**77.69**	82.31	76.46	**2.16**	**108**	**0.46**
		Relief	61.75	69.23	60.86	3.23	135	0.69
		mRmR	57.87	63.79	53.18	4.27	124	0.51
		GA	69.56	71.88	66.81	2.26	139	0.81
**SVM**	ColonTumor	Full Set	73.86	80.63	70.66	3.98	2000	‒
		FSBRR	**87.68**	91.46	83.21	**3.48**	**34**	**1.12**
		Relief	80.46	84.18	75.44	4.23	38	3.22
		mRmR	81.78	85.71	75.81	4.58	42	3.96
		GA	84.16	89.66	81.62	4.40	48	28.86
	Nervous-System	Full Set	53.34	65.15	50.86	6.58	7129	‒
		FSBRR	**73.18**	78.12	70.69	**3.28**	**37**	**8.78**
		Relief	68.56	72.61	62.81	3.92	44	17.89
		mRmR	65.78	72.48	61.15	5.02	42	19.96
		GA	68.16	72.11	63.56	3.52	45	42.11
	DLBCL-Stanford	Full Set	70.16	79.21	60.79	5.86	4026	‒
		FSBRR	**78.91**	83.45	74.41	4.31	**29**	**2.51**
		Relief	72.96	74.61	62.69	5.95	45	4.41
		mRmR	72.15	77.65	69.15	**3.82**	54	3.25
		GA	72.85	77.45	68.06	3.85	91	12.54
	p53 Mutants	Full Set	85.56	87.76	80.13	4.11	5409	‒
		FSBRR	**86.25**	90.02	82.15	3.11	39	**2.35**
		Relief	84.15	87.26	80.36	**3.09**	54	2.85
		mRmR	82.15	87.58	76.05	4.85	**37**	3.91
		GA	84.55	88.12	78.51	3.98	61	20.51
	Arcene	Full Set	70.56	74.15	66.58	3.55	10000	‒
		FSBRR	**80.61**	84.52	78.15	**3.54**	**51**	**5.55**
		Relief	76.59	80.12	70.54	3.85	73	8.52
		mRmR	74.75	79.65	70.25	4.65	71	8.15
		GA	71.35	75.85	65.35	4.05	115	55.85
	BRCA	Full Set	77.25	80.24	74.21	3.17	21548	‒
		FSBRR	**82.02**	86.55	80.85	**1.80**	**148**	**7.56**
		Relief	79.05	85.89	76.19	3.29	241	8.58
		mRmR	78.49	86.01	71.71	5.85	189	9.77
		GA	79.96	86.36	75.45	3.25	262	8.40
	GBM	Full Set	65.12	71.55	61.10	3.40	18348	‒
		FSBRR	**80.09**	85.55	77.35	**2.01**	61	**6.45**
		Relief	74.01	78.23	71.08	2.75	**53**	7.88
		mRmR	73.99	79.00	70.59	3.44	68	8.58
		GA	80.03	87.10	76.40	3.36	101	9.51
	TSP	Full Set	64.45	72.14	60.45	5.84	319	‒
		FSBRR	**72.35**	76.18	69.98	**2.25**	**108**	**0.42**
		Relief	62.65	67.18	58.10	3.94	135	0.70
		mRmR	56.90	61.54	51.95	4.75	124	0.52
		GA	64.67	72.09	60.46	5.18	140	0.75

Note: (1) full set: a set of all features that have not been processed by feature selection algorithm. (2) Mean (%): the mean of performance. (3) Max(%): the highest of performance. (4) Min(%): the lowest of performance. (5) Std: the standard deviation. (6) MeanFN: the mean number of selected feature. (7) RT(s): running time, unit is second.

Boldface indicates the best experimental result.

From Table 4, we can observe the following aspects: (1)The FSBRR algorithm obtained the highest Mean among all the four feature selection algorithms for twenty-one out of twenty-four experimental results. The highest Mean(s) were 92.01%, 80.17%, 82.99%, 94.31%, 85.67%, 86.26%, 78.63%, 91.91%, 74.24%, 83.68%, 85.67%, 84.42%, 77.69%, 87.68%, 73.18%, 78.91%, 86.25%, 80.61%, 82.02%, 80.09%, and 72.35% respectively. Meanwhile, we notice that the maximum Mean improvement of FSBRR was 19.84% compared with the full set. (2) RF-based GA algorithm uses GBM dataset, and KNN-based GA algorithm uses p53 Mutants and GBM datasets to obtain highest Mean of 82.03%, 89.90%, and 81.39%, respectively. However, Std, MeanFN and RT of GA were significantly higher than FSRRR in these three experiments. (3) The Std among for eighteen out of twenty-four experimental results obtained by FSBRR was smaller than other three algorithms. (4) Four feature selection algorithms can effectively reduce the feature dimension, but the dimension reduction of the FSBRR algorithm was the most obvious. In FSBRR, Relief, mRmR and GA, GA belongs to the wrapper feature selection algorithm, so there were differences in the number of feature subsets for RF, KNN and SWM classifiers. (5) In most experiments, except for the running time index, the other performances of RF were significantly better than the KNN and SVM for the same dataset. Such results indicate that for the specified dataset, to get the best experimental results, a matching classification (or learning) algorithm must be found.

**Fig 1:**
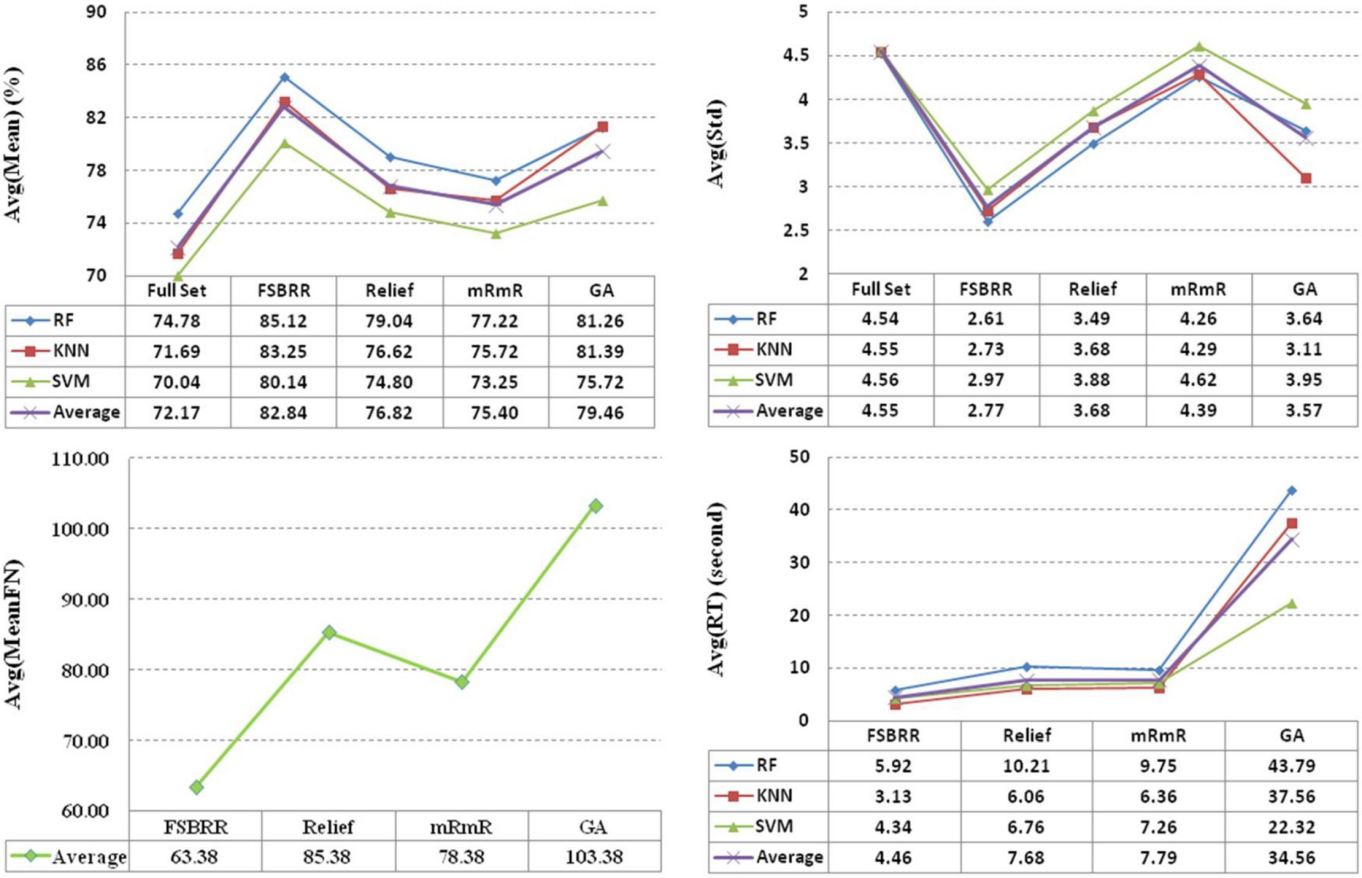
The average attributes value of four feature selection algorithms based on eight datasets.

Fig 1 was obtained by statistical analysis of Table 4. It shows four average attribute values (avg(Mean), avg(Std), avg(MeanFN), and avg(RT)) of four feature selection algorithms. From Fig 1 we can observe that performance, stability, the feature number of optimal subset, and time complexity of FSBRR was superior to the other three algorithms. We believe that the reasons for obtaining this experiment were: (1) FSBRR algorithm uses *R*_*i*,*j*_ to explore the horizontal correlation between features and features, and the longitudinal correlation between features and classes was explored by *R*_*i*,*c*_. Meanwhile, based on the basic relevance theory, the horizontal relevance and the longitudinal relevancewere effectively combined. (2) FSBRR algorithm not only removes the irrelevant features, but also removes the approximately redundant features.

## Discussion and analysis

**Fig 2:**
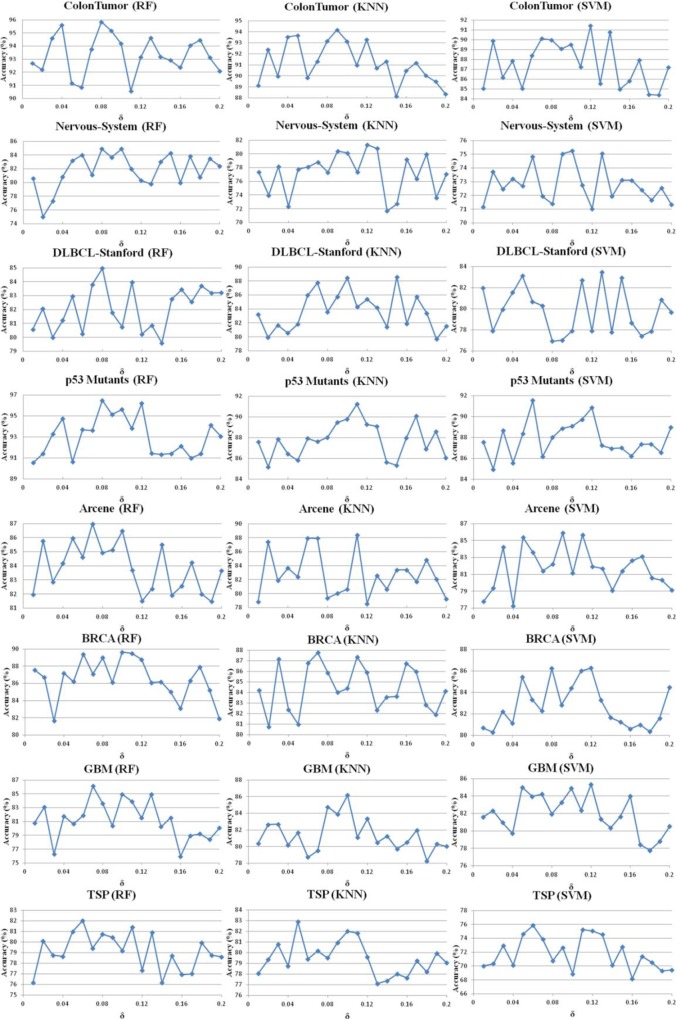
The relationship between parameter δ and classification accuracy.

**Fig 3:**
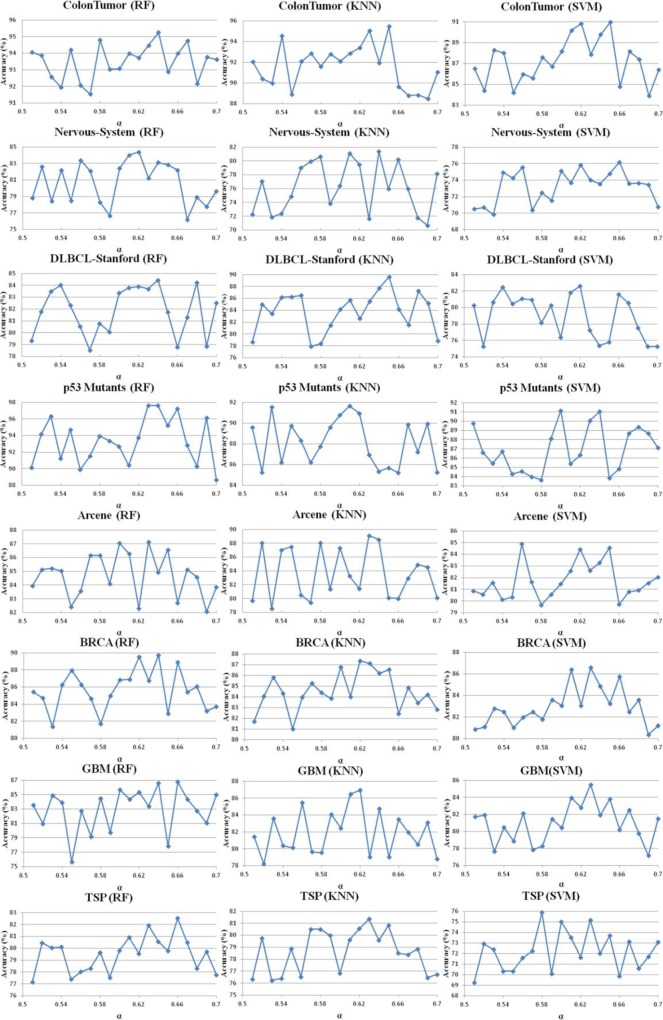
The relationship between parameter α and classification accuracy.

**Fig 4:**
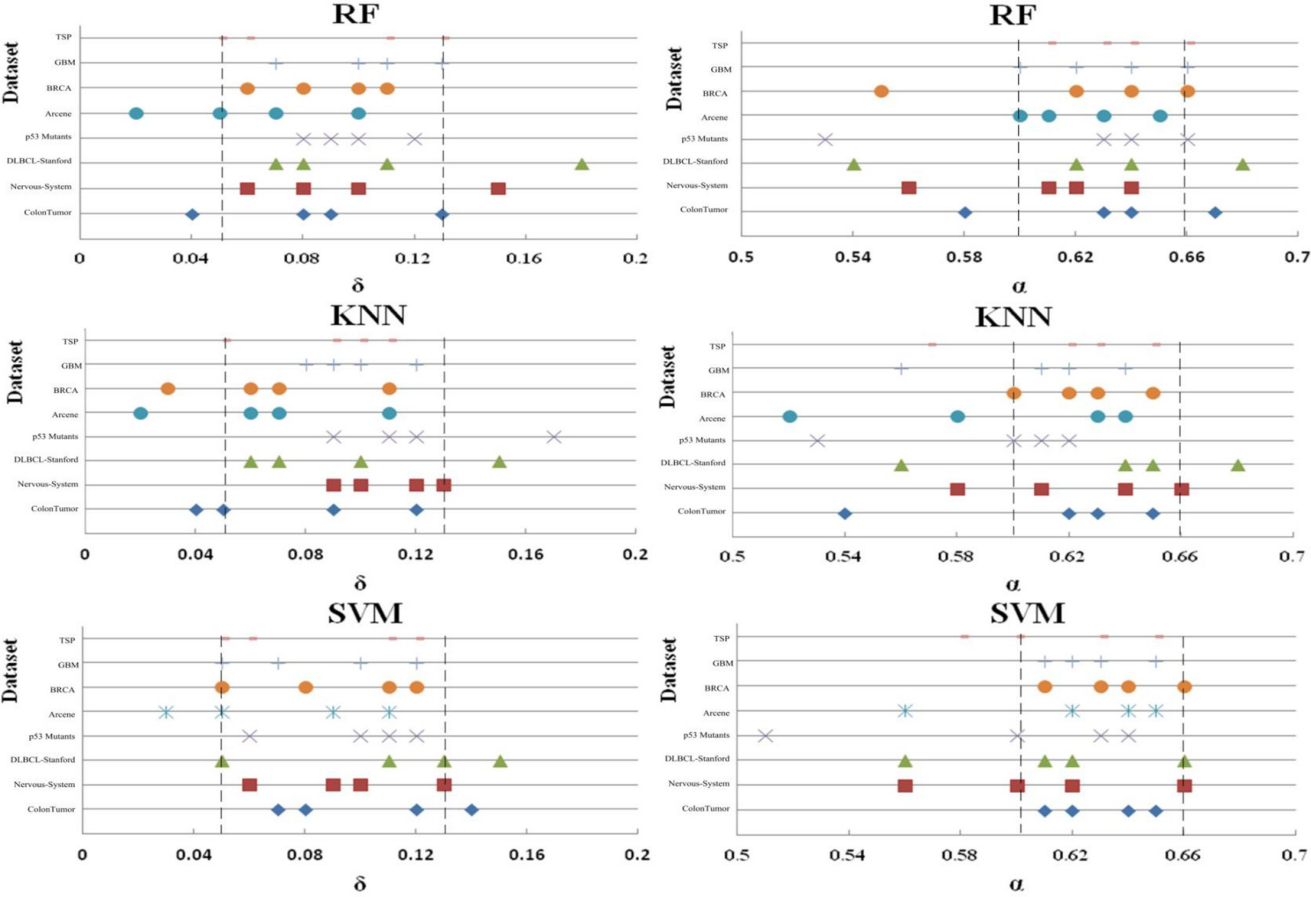
The range of parameter values when the classification accuracy is located in the top 20% based on FSBRR algorithm.

To verify the effect of parameters δ and α on the performance of FSBRR algorithm, the classification accuracy of 8 datasets was tested on RF, KNN, and SVM, respectively. In the first experiment, when the parameter α = 0.6 keep constant, the parameter δ increased from 0 to 0.2 with a step length of 0.01. In the second experiment, when the parameter δ = 0.1 keep constant, the parameter α increased from 0.5 to 0.7 with a step length of 0.01. Other experimental procedures are described in experimental design section. Moreover, to facilitate discussion and analysis, the classification accuracy was only considered in here. The results of the two experiments were shown in Figs 2 and 3. Statistical analysis of the experimental results in Figs 2 and 3 reveals that: when the highest classification accuracy was obtained for eight datasets, although the values of parameters were different, their ranges of value were overlapped. By selecting the results of the top 20% of the classification accuracy, we can clearly observe that the saliency overlap of the parameter ranges, and the results were shown in Fig 4. The optimum range of parameter δ is [0.05, 0.13], and the optimum range of parameter α is [0.60, 0.66]. See the dotted line marker range in the Fig 4.

The approximate optimal feature subset of each original dataset may be 1 or more. The purpose of feature selection is to find one of the optimal subset. However, the dimensions of the optimal subset for different original datasets are different, and the correlation values (*R*_*i*,*j*_ and *R*_*i*,*c*_) distribution of different datasets is vary, which leads to the difference of parameter values (δ or α). According to the statistical analysis of the above two experimental results, the parameters δ and α should be selected within the range in [0.05, 0.13], and the range in [0.60, 0.66], respectively. Because the classification accuracy may be reach the highest value in this range.

In order to further analysis and interpretation of our proposed algorithm, a Breast cancer Wisconsin (Diagnostic) [42] dataset from UCI was used in this subsection. This dataset contains two kinds of data: malignant and benign. It was composed of 569 instances, and each instance contains 32 attributes. For FSBRR algorithm, the relationship between relevance and redundant features was analyzed using RF algorithm. Other experimental procedures are described in experimental design section. Besides, to facilitate discussion and analysis, the classification accuracy was only considered in here.

The main results of this experiment were listed in Table 5. For Table 5 there were two attributes need to be explained which are: (1) "Feature set", for example, {5} was the subscript of the feature, which is the fifth feature. (2) "Change" was the accuracy change based on the current feature set. From Table 5 we can observe the following points: (1) {5} as the current feature set, the accuracy rate increases after adding feature 7 (see the third row), in contrast, the accuracy rate reduces after adding feature 21 (see the fourth row). These results verify the redundancy **Criterion 1** (when *R*_*i*,*j*_ is large, whether *F*_*i*_ is redundant is uncertain). In this case, the approximate redundant feature definitionis needed to determine whether the current feature is redundant. (2) {5, 7} as the current feature set, the accuracy rate increases after adding feature 18 (see the fifth row); {8, 3} as the current feature set, the accuracy rate increases after adding feature 30 (see the seventh row). These results verify the redundancy **Criterion** 2 (when *R*_*i*,*j*_ is smaller, no matter the size of the *R*_*i*,*c*_, the feature *F*_*i*_ is not redundant).

**Table 5:** The relationship between relevance and redundant feature based on FSBRR algorithm.

**Feature set**	**Accuracy (%)**	**Change**	*R*_**i,c**_	*R*_***i*,*c***_-value	*R*_*i*,*j*_	*R*_*i*,*j*_**-value**
{5}	83.30	‒	*R*_*5*,*c*_	0.9157	‒	‒
{5,7}	87.12	↑	*R*_*7*,*c*_	0.9011	*R*_*5*,*7*_	0.9454
{5,21}	83.16	↓	*R*_21,*c*_	0.8045	*R*_*5*,*21*_	0.9101
{5,7,18}	87.64	↑	*R*_18,*c*_	0.4562	*R*_*5*,*18*_ */R*_*7*,*18*_	0.2131/0.1983
{8,3}	84.50	‒	*R*_8,*c*_ */ R*_*3*,*c*_	0.8934/0.9013	‒	‒
{8,3,30}	87.86	↑	*R*_30,*c*_	0.7120	*R*_*8*,*30*_ */R*_*3*,*30*_	0.3010/0.2139

## Conclusions

In this paper, the relationship between two kinds of correlation (*R*_*i*,*c*_ and *R*_*i*,*j*_) is established, which effectively combines the correlation between features and classes and the correlation between features and features to eliminate redundant features. Because the determination of completely redundant features in actual operation is difficult to realize, so we first analyze four kinds of boundary conditions between *R*_*i*,*c*_ and *R*_*i*,*j*_, and then a redundancy feature criteria is proposed. On this basis, the approximate redundancy features are defined in this study. Finally, we have proposed a new feature selection algorithm based on approximate redundancy removal (FSBRR) for high dimensional biomedicine data classification.

To verify the effectiveness of the FSBRR algorithm, three classification algorithms (RF, KNN and SVM) are used to compare the FSBRR and three typical feature selection algorithms on eight high dimension biomedical datasets. The experiment results show that the FSBRR algorithm can effectively remove redundant features to improve the classification performance. Additionally, we also designed a set of comparative experiments to discuss and analyze the effects of parameters δ and α on the performance of FSBRR algorithm.
